# Balancing Technology, Pedagogy and the New Normal: Post-pandemic Challenges for Higher Education

**DOI:** 10.1007/s42438-021-00249-1

**Published:** 2021-08-09

**Authors:** Chrysi Rapanta, Luca Botturi, Peter Goodyear, Lourdes Guàrdia, Marguerite Koole

**Affiliations:** 1 Universidade Nova de Lisboa, Lisbon, Portugal; 2 University of Applied Sciences and Arts of Southern Switzerland, Locarno, Switzerland; 3 The University of Sydney, Sydney, Australia; 4 Universitat Oberta de Catalunya, Barcelona, Spain; 5 University of Saskatchewan, Saskatoon, Canada

**Keywords:** Emergency remote teaching, Online learning and teaching, Higher education, Post-Covid, Pedagogy, Digital technologies

## Abstract

The Covid-19 pandemic has presented an opportunity for rethinking assumptions about education in general and higher education in particular. In the light of the general crisis the pandemic caused, especially when it comes to the so-called emergency remote teaching (ERT), educators from all grades and contexts experienced the necessity of rethinking their roles, the ways of supporting the students’ learning tasks and the image of students as self-organising learners, active citizens and autonomous social agents. In our first *Postdigital Science and Education* paper, we sought to distil and share some expert advice for campus-based university teachers to adapt to online teaching and learning. In this sequel paper, we ask ourselves: Now that campus-based university teachers have experienced the unplanned and forced version of Online Learning and Teaching (OLT), how can this experience help bridge the gap between online and in-person teaching in the following years? The four experts, also co-authors of this paper, interviewed aligning towards an emphasis on pedagogisation rather than digitalisation of higher education, with strategic decision-making being in the heart of post-pandemic practices. Our literature review of papers published in the last year and analysis of the expert answers reveal that the ‘forced’ experience of teaching with digital technologies as part of ERT can gradually give place to a harmonious integration of physical and digital tools and methods for the sake of more active, flexible and meaningful learning.

## Balancing Technology, Pedagogy and the New Normal: Post-Pandemic Challenges for Higher Education

The Covid-19 pandemic has presented an opportunity for rethinking assumptions about education in general and higher education (HE) in particular (Ashour et al. 2021; Jandrić et al. 2020; Peters et al. 2020). Although visions for the future of HE vary and are contested, there is a growing consensus that ‘nothing could be worse than a return to normality’ (Roy 2020). Notwithstanding the lack of preparation and difficulties faced by teachers, educational administrators and institutions, the overall picture now reveals an openness towards innovation and new learning opportunities that were not as evident before. In the light of the general crisis the pandemic caused, especially when it comes to the so-called emergency remote teaching (ERT) (Hodges et al. 2020; Xie and Rice 2021), educators from all grades and contexts experienced the necessity of rethinking their roles, ways of supporting students’ learning tasks (Rodríguez-Triana et al. 2020; Nordmann et al. 2020) and the image of students as self-organising learners, active citizens and autonomous social agents (Council of Europe 2016, 2018). This shift requires new learning, not only for students but in particular for teachers. As Hollander (2021) intelligently remarks, ‘the pandemic is taking higher education back to school’.

Although ERT has little in common with online learning and teaching (OLT) and even less with Internet-based distance education, they share one common feature, which is teaching with digital technologies. As Xie and Rice (2021) nicely summarise, ERT and OLT do not share the same definition, goal, design process, instructional delivery mode or ways to integrate technology. On the one hand, ERT refers to a temporary shift due to crisis circumstances (Hodges et al. 2020): its goal is to provide ‘a reliable, temporary, fast, and durable access’ to instruction and its affordances (Mohmmed et al. 2020: 2); no time for the actual preparation of online activities and materials is foreseen, and this lack of support and resources, including lack of time, resulted in mostly synchronous class meetings (Manfuso 2020).

On the other hand, OLT is a subset of distance education using electronic media that, if done well, takes place in dynamic and carefully designed learning environments (Keengwe and Kidd 2010: 533–534). It provides a well-considered learning ecosystem (Manfuso 2020), aimed at increased flexibility and better access to learning opportunities, through the careful design of unique courses that appropriately combine synchronous, asynchronous and independent study activities (Anderson 2008). In its turn, distance education, and in particular Internet-based distance education, refers to ‘an institution-based, formal education where the learning group is separated, and where interactive telecommunications systems are used to connect learners, resources and instructors’ (Schlosser and Simonson 2009: 1). According to Garrison (2009), both traditional (i.e. campus-based) and distance HE have been assisted by OLT practices to innovate their instructional paradigm and approach, given the focus of OLT on active, collaborative, social-constructivist processes. This distinction between OLT and distance education is useful in order to understand when ERT implementation is only based on the in-person versus online teaching mode shift, or when instead it also embraces a shift from expository to interactive teaching and learning (Moorhouse and Kohnke 2021).

Another consideration to take into account is that the emergency shift to online teaching overemphasised its *remote* qualities, arising from the need to avoid in-person interactions due to the pandemic restrictions. However, other aspects of technology-based teaching, which to a higher or lower degree were also present in traditional university practices, were underemphasised, probably because they were not as integrated in existing campus-based teaching as they could, or should, have been. These include aspects of blended learning (Garrison and Kanuka 2004) and flipped classroom activities (O’Flaherty and Phillips 2015), in which the in-class time is combined with productive learning outside the classroom. Such aspects could easily be incorporated into exclusively online interactive instruction, replacing the ‘in-class’ time by dynamic synchronous activities (including small-group discussions) and the ‘outside-class’ time by group asynchronous activities and individual assignments. Nonetheless, the pre-Covid-19 level of digitalisation of HE systems and underdevelopment of pedagogical strategies did not allow for such a smooth shift to take place. Instead, the shift from face-to-face to ERT was often perceived as *disruptive* (Iglesias-Pradas et al. 2021; Wyatt-Smith et al. 2021), *aggressive* (Watermeyer et al. 2021), *disastrous* (Dhawan 2020) and *unwelcome* (Watermeyer et al. 2021).

The diversity of reactions to the implementation of ERT was the inspiration for our first *Postdigital Science and Education* paper (Rapanta et al. 2020), in which we sought to distil and share some expert advice for campus-based university teachers to adapt to online teaching and learning. Now, 1 year later, there is a second round of mixed reactions, varying on a continuum between ‘Finally back to before, let’s forget this nasty period’ and ‘Here are the lessons we learned’ and that could help to enhance our educational offer in any version of the future. As Anderson (2021: 3) notes, the concept of ‘crisis’ can be used by those in power, and those seeking to protect their vested interests, to force people to make choices between diametrically opposed alternatives, such as ‘on-campus’ versus ‘online’. This obscures more nuanced and integrative choices. The goal of this paper is to expand our collective ‘room for manoeuvre’ (Anderson 2021: 4) by providing a synthesis of well-informed learning design suggestions and reflections for campus-based and digitally enhanced university teaching in the post-Covid era.

## Literature Review

The Covid-19 pandemic and its subsequent lockdown phases spurred the need for focusing beyond routines and understanding teachers’ role(s) as active and creative agents, negotiators and integrators of digital and pedagogical resources into meaningful teaching–learning practices (Damşa et al. 2021). A large number of studies in the last year have therefore looked at how teachers reacted to the urgent shift from face-to-face to online teaching and whether this transition has brought about positive changes in their implicit and explicit pedagogical models and strategies (Jandrić et al. 2020; Peters et al. 2020; Sangrà et al. 2020).

An interesting study was carried out in Norway, a country which occupies a leading position in terms of promoting educational digital infrastructures, but also faces a low digital competence among its educators (Damşa et al. 2021). The researchers administered an online survey to 171 university teachers, asking about their experiences, challenges and perceived effects of the online transition on learners, during the first month of teaching remotely due to the Covid-19 lockdown. Three teacher profiles emerged from the quantitative analysis according to whether teachers showed a low (Profile 1), medium (Profile 2) or high (Profile 3) use of new online teaching methods, software and support from others they found useful. The qualitative analysis matched Profile 1 teachers (36.7% of the participants) with a tendency towards iterative, non-transformative agency where activity in emerging contexts replicated existing practices. Profile 2 teachers (55.2% of the participants) were more prompt to show a practical-evaluative type of agency in which teachers acknowledge the use(fulness) of technologies as alternatives to their ordinary practice, but not their potential as triggers for new practices. Finally, Profile 3 teachers (only 8% of the participants) showed evidence of future-projective, transformative agency.

Scherer et al. (2021) conducted a large-scale survey to which 1144 (afterwards restricted to 740) educators from 64 countries replied, with a large majority (more than 80%) being from European universities. The survey was an adaptation of the validated T-PACK self-efficacy scale (Archambault and Crippen 2009) to the context of online teaching and learning. In particular, it aimed at identifying the pedagogical and content-related aspects of university teachers’ online teaching readiness, such as their confidence in technology-based content knowledge (TCK; e.g. implementing curriculum in an online environment), technology-based pedagogical knowledge (TPK; e.g. implementing different methods of teaching online) and technology-based pedagogical content knowledge (TPCK; e.g. using technology to predict students’ skills or understanding of a particular topic).

They identified three profiles describing university teachers’ readiness for online teaching, namely (a) a low readiness profile, with teachers rating low on both personal and contextual readiness indicators; (b) an inconsistent readiness profile, with low ratings of TPCK self-efficacy and perceived online presence, but high ratings on perceived institutional support; and (c) a high readiness profile with consistently high ratings on all readiness indicators. Membership in one profile or another was shown to be dependent on a variety of characteristics such as prior online teaching experience, the number of days spent for preparation for the online teaching shift and the number of days into online teaching after the shift. These characteristics suggest that the more immersed teachers were in the online teaching and learning experience, the readier they felt about making the shift.

Daumiller’s et al. (2021) study in Germany also used online surveys but combined teachers’ and students’ responses. In total, 80 academics replied to a survey with the majority (more than 80%) having no or little previous experience with online teaching. In addition, 703 students attending their courses also replied to a survey. The teachers’ survey focused on their instructional achievement goals, defined as: learning approach (e.g. ‘I want to constantly improve my competences’), performance (appearance) approach (e.g. ‘I want to be perceived as competent’), performance (appearance) avoidance (e.g. ‘I want to avoid being perceived as incompetent’) and work avoidance goals (e.g. ‘I want to have as little to do as possible’). Faculty attitudes towards the sudden shift to online teaching were assessed as being a perceived threat, a perceived positive challenge or a perceived opportunity for competence development.

Overall, the analysis revealed higher means for perceived positive challenge and perceived usefulness for competence development than for perceived threat, suggesting that participating teachers’ attitudes towards the change were in general more favourable than unfavourable. In addition, the teachers’ learning approach goals were positively associated with perceiving the shift to online teaching as a positive challenge and opportunity, whereas performance (appearance) avoidance and work avoidance goals were associated with perceiving this change as threatening. This latter attitude, perceived threat, was also related to higher burnout levels and to students’ lower ratings of teaching quality.

The findings regarding university teachers’ preparedness and welcoming of the shift to online teaching expand beyond Europe. Marek et al. (2021) conducted a mixed-method survey study with 413 faculty participants, the majority (90.2%) being from Asia. In contrast to the previous study by Daumiller et al. (2021) where the large majority of participants did not have any previous experience with online teaching, almost half of the participants (46.9%) in the Marek et al. (2021) study said that they had used online technologies (other than PowerPoint or discipline-specific software) in their classes before the pandemic. What is more, this previous experience, when present, predicted the ease and comfort with which the participants shifted to online teaching during the pandemic.

Another interesting finding was that less than half of the participants used a university-provided learning management system, with the majority opting for a wide range of other technologies available online. Finally, in their open-ended answers, respondents emphasised the need for adaptability and good planning, revealing an attitude of ‘doing what it takes’ to serve their students under the circumstances. Adding to these findings, a study by Ashour et al. (2021: 12), using an online survey with almost a hundred HE experts (university managers and professors) in the United Arab Emirates, revealed that almost all respondents were confident that online learning was ‘here to stay and could make a much stronger contribution to higher education in the years ahead’.

When it comes to students, results were mixed as well, with studies either pointing at the dark or the bright side of the universities’ shift to ERT. An example of the ‘dark side’ is presented by Daniels et al. (2021), who report the results of a survey with 98 undergraduate students of different disciplines at Canadian universities. The survey aimed at revealing (1) students’ achievement goals, defined as mastery approach, mastery avoidance, performance approach and performance avoidance goals; (2) their behavioural, emotional and cognitive engagement; and (3) their perceptions of cheating and success (note: although for ‘1’ and ‘2’ two multiple-item validated scales were used, ‘3’ was assessed through the use of just two Likert-scale items added by the researchers). Students gave their self-reported answers to the survey items with regards to two conditions: a past condition, referring to the semester before ERT was implemented, and the present (at the time of the survey) condition referring to the first semester ERT was implemented.

The analysis showed that students’ achievement goals, engagement and perceptions of success all significantly decreased during the ERT semester, while their perceptions of cheating increased. Similar results are presented by Aguilera-Hermida (2020) who conducted a mixed-method study with 270 students from North American universities, aiming at revealing their attitude, affect and motivation towards the educational delivery method, their perceived behavioural control and their perceived challenges and positive aspects during the Covid-19 period. Her results clearly indicate that the transition to remote learning was an unpleasant experience for the majority of the student respondents, who claimed that learning online was more difficult and less motivating than face-to-face, mainly because of limited access to resources to finish their assignments and lack of communication with their professors. The positive aspects reported were not related to the educational experience but to the fact of spending more time at home and less time at school.

In contrast to the negative findings of Daniels et al. (2021) and Aguilera-Hermida (2020), there is research confirming that the academic performance of students during the Covid-19 confinement *improved* as compared to previous years. For instance, Iglesias-Pradas et al. (2021) compared the academic results of Telecommunications Engineering students in Spain during the Covid-19 pandemic with those of previous years, using both quantitative (academic records across 43 undergraduate courses) and qualitative (open-ended questions to course coordinators) data. They found a significant increase in students’ performance during the first year of the pandemic (2019–2020), as compared to the previous 2 years. They attribute this positive result to organisational factors, such as the high level of preparedness of the educational institution in terms of technical infrastructure, the existence of informal communication channels among faculty and administrators and the semi-decentralised structure of the institution which allowed instructors to quickly make decisions on the tools, design and strategies to use.

Adding to this, in a quantitative large-scale study in India with 544 student respondents, Gopal et al. (2021) showed a positive correlation between quality of instructor, course design, prompt feedback and students' expectation, on the one hand, and students’ satisfaction and performance, on the other. Survey data in Australia revealed mixed experiences. Students reported that they valued the increased opportunities for managing their own time and the wider range of assessment methods made available to them. There was also evidence of improved academic results. Students reported negatively when they had technology-related problems, where access to teaching staff was restricted and where teachers showed that they had insufficient expertise in using digital instruments (Martin 2020).

The ERT implemented during the Covid-19 pandemic also brought up some contradictions which were not evident before: the social distancing between students and teachers, among students, and also among teachers, was contrasted with the increased accessibility facilitated by the virtual learning spaces (Lau 2021); the burnout and distress of both teachers and students was accompanied by an increased flexibility and positive dialogic relationships implemented by several educators as a strategy to cope with and anticipate their students’ drop out (Thierauf 2021); and the lack of resources and administrative support for faculty to (re)design their courses was balanced with evidence of increased creativity and responsiveness by both teachers and students alike (Chemi 2020; Cramman et al. 2021).

The most salient negative aspect directly related to the ERT was the way it sharply revealed socioeconomic gaps, prejudicing all those students who lacked sufficient bandwidth, whose families lacked enough devices for everyone to use, who were unable to find appropriate study spaces at home or whose financial needs during the pandemic forced them to increase time spent at work, at the expense of time spent studying (Jandrić et al. 2020; Peters et al. 2020; Schatzki 2021). The extent of these economic and digital inequalities (Murphy 2021) became much more obvious — as did the fact that so-called digital natives are not necessarily capable digital learners (Iglesias-Pradas et al. 2021).

## The Present Study

The difficulties experienced by teachers and learners, summarised above, led us in 2020 to investigate the different ways in which educators can support their students in the transition to online learning, as well as the different learning design strategies they can implement to be able to teach online (Rapanta et al. 2020). This need was born due to an observed overemphasis on the digital aspects of OLT, as opposed to the pedagogical knowledge accompanying digital competence (Kali et al. 2011). Our 2020 study shed light on what Garrison and Kanuka (2004) and Anderson (2008) had claimed several years ago, namely that online and face-to-face teaching share the same values and require the same quality of teacher presence and support when it comes to monitoring learning processes. What is more, through our expert interview analysis, we noted that learning design skills and activities, commonly reported as part of the online teacher pedagogical knowledge toolkit, are relevant for any type of university teaching, as they increase the opportunities for high-quality learning.

In the present study, we ask the following question: Now that campus-based university teachers have experienced the unplanned and forced version of OLT, how can this experience help bridge the gap between online and in-person teaching in the following years? This question is relevant for several reasons. First, as Iglesias-Pradas et al. (2021) observe, the unplanned nature of ERT is dissimilar to other transitions from face-to-face to blended, online or flipped teaching in the past, which may have already been part of the strategic plan(s) for innovation of several campus-based HE institutions. Therefore, it is possible that negative perceptions and fear regarding OLT were formed on the part of both teachers and students *due to* their experiences of ERT disguised as OLT. This negative experience can lead to an overstated necessity of ‘going back to normal’ without asking whether the pre-Covid instructional practices in many face-to-face lecture scenarios in HE, including expository, monological and passive chalk-and-talk, can be considered ‘normal’ or desirable at all.

Second, according to evidence reported in the pre-Covid era, the uptake of online and blended learning by HE institutions has increased significantly in recent years (Conrad and Openo 2018; Power 2020). If this is true, and we do not have a reason to believe to the contrary, why did the pandemic catch so many off guard? A possible explanation is that the ‘institution’ is probably not the right unit of analysis. In many cases, distance or online education offers are limited to one program or even one course. This implies that the infrastructure remains limited and that the required design and delivery OLT competences rest only with a few teachers. This resembles the ‘Lone Ranger’ approach depicted by Tony Bates (2000) as the most common model of e-learning course development. Lone Rangers — i.e. innovators, pioneers — are ‘essential for getting innovation started, for demonstrating the potential of technology for teaching, and for ensuring e-learning is used when there is no systematic support from the institution’ (Bates 2004: 285), but their efforts are seldom enough to make a sustainable difference on a large scale.

## Method and Findings

The goal of this paper is to offer reflections for more innovative teachers to emerge in post-Covid campus-based institutions, and possibly make their efforts mainstream within their organisation. The shift marked by the pandemic’s ERT can turn into an opportunity—as long as OLT design principles and competences are applied. In the 2020 paper, we asked about the basic learning design pedagogical knowledge university teachers must have to be able to teach in an online setting. Now, 1 year later, we seek to consolidate that knowledge in a package of expert advice regarding possible future steps that may change the landscape of university teaching capitalising on the ERT experience.

We decided to follow the same method and format as in our 2020 paper. In particular, the method used for this study was again expert interviews (Bogner et al. 2009), which was considered appropriate for the emerging and urgent topic of (rein)forced digitalisation of teaching and learning during and after the Covid-19 pandemic (Jandrić 2020). The four participants were selected according to their proven expertise and deep experience in the field of online teaching and learning (for details about the criteria for their selection, see Rapanta et al. 2020). These experts are also co-authors of the paper, and their answers to the interview questions are co-presented as if they formed part of the same discussion panel (see Asterhan et al. 2020, for a similar format). The interviews included five questions and were administered by e-mail, after the goal of the research was explained to the participants. The four experts were blind to the responses of each other, until a first complete draft of the paper was presented to them, to which they all contributed with comments and revisions.

The interview questions were the following:

1. What is the role that digital technologies and distance learning can play in campus-based education after the Covid-19 experience?
2. How might university teachers think about themselves and their role now? How do you think their identity has changed/must change?
3. How can learning assessment change in the post-Covid era?
4. What advice would you give to campus-based university teachers interested in improving their students’ self-regulation skills? What types of activities can be designed towards this direction?
5. Overall, what are some lessons that distance/online learning can teach to campus-based university teachers?

Questions 1 and 2 are general questions informed by the current contradictory situation, in which universities around the world are urged to shift back to face-to-face teaching, with some hybrid solutions applied, when necessary, while research evidence inclines towards the effective blending and co-existence of online and face-to-face teaching practices, mixed according to teachers’ and students’ needs. Questions 3 and 4 draw on the findings of Rapanta et al. (2020), where assessment methods and students’ self-regulation skills emerged as central themes in the expert interviews. Finally, question 5 functions as a knowledge consolidation question, inverting the scope of Rapanta et al. (2020): previously we asked how campus-based teachers can transform themselves into online teachers amplifying their teaching presence; now we ask how online teaching can become a permanent learning resource for campus-based HE practices in the post-Covid era.

To facilitate readers’ connection between the interview answers and their discussion we marked the key idea(s) in each answer in italics.

### Question 1: What is the Role that Digital Technologies and Distance Learning can Play in Campus-based Education after the Covid-19 Experience?

#### Luca Botturi (E1)

The Covid-19 experience pushed the digitalization of teaching and learning in HE: teachers discovered that they *can* teach online, managers invested in infrastructure and support services, students adjusted their practices and expectations. Most teachers and students adjusted to the new situation, but also understood that online distance learning, at least in the ERT experience they had, is not the best option and is not equal to campus-based education. For example, informal and group interactions are heavily reduced, and evaluation is less fair (and it’s easier to cheat). Nonetheless, it has original affordances: flexibility in organization, less travelling, possibility to view lectures more than once, etc.

I think the goal now is *keeping what works and blending it seamlessly* with campus-based education. This does not depend on the tools (which are now in place) and on individual skills, rather on *academic strategy* (what was pioneering or emergency should become mainstream), *proper curriculum and course design*, including key decisions about synchronous/asynchronous learning, evaluation practices, etc.

The key criteria for developing such a strategy comes from the answer to one easy question: What is the real added value of meeting in person? Is it the campus experience? Is it attending to classes where students are active? Is it the resources I can access? Answers to these questions are different for different universities and might be different for different student groups.

#### Peter Goodyear (E2)

I think we have to start by acknowledging that developments associated with ERT have changed conceptions about how established working practices in teaching can become unsettled. What once looked like the only or the most natural patterns for teaching and learning suddenly come into question, and other patterns emerge as possible and sometimes beneficial. In part, I think this change is to do with the barely acknowledged reliance of educational practices on physical infrastructure. Change the infrastructure, and many elements of existing practices that seemed tightly bound together suddenly free up. Associated with this, we can also see that university teachers and university leaders are *struggling to find language and concepts with which to discuss what should and should not be done*, going forward. So, for instance, what do ‘campus-based’, ‘online’ and ‘blended’ actually mean? *What distance is being measured in ‘distance learning’*? Some teachers are saying how Zoom classes, for example, are much more ‘face-to-face’ than is the case with on-campus lectures.

I think one of the changes that will have considerable effects is that students now realise that it is not so difficult for universities to operate in a much more flexible fashion, and that *they are actually able to fit educational offerings around students’ lives,* rather than requiring students to fit their lives around study. Given the very substantial numbers of university students who are more mature (not ‘straight from school’), who have family and job commitments, who need more flexible patterns of study, I think we can see this as an important change. In short, I think student demand for more flexible forms of educational provision will mean a continuing, and expanding, role for digital technologies and the approaches pioneered in open, flexible and distance education.

#### Lourdes Guàrdia (E3)

There is no doubt that Covid-19 is accelerating digital transformation in the education sector. Campus-based education is adopting new models of teaching and learning, where the use of ICT is playing a central role. Teaching and learning online or intensively supported by technologies will be combined with face-to-face models, and probably the use of technology will be broadly considered in all programs as a strategic and essential aspect, no longer being ‘just face-to-face’. In this sense, *the idea of a blended learning and flipped classroom approach adoption* that has been promoted and researched in recent years as highly effective will probably prevail over face-to-face models, where technology is not relevant at all.

#### Marguerite Koole (E4)

It is still too soon to know the full efficacy of the Covid-19 vaccines, so the next few years will remain uncertain. Therefore, it is wise to remain amenable to digitally mediated modalities. Blended strategies may facilitate flexibility when needed. Alternative means of access to face-to-face classes are most likely to involve the download of readings, exercises, and activities as well as a mixture of synchronous or asynchronous means of attending lectures. *The very meaning of the word ‘attendance’ has already started shifting beyond the physical, reflecting instead, a variety of ways of accessing and interacting with content, fellow students, and instructors*.

‘Attendance’ is increasingly aligned with Garrison et al. (2001) concept of presence as per the community of inquiry (CoI) model. The Oxford English dictionary provides several definitions of presence, most of which refer to the physical; however, there is also a sense that it applies to the non-physical: ‘A person or thing that exists or is present in a place but is not seen.’ (OED 2021). This definition can be usefully applied to the digital. As such instructors would benefit from expanding their conceptions of class attendance to include a greater array of evidence for interaction and understanding. In this way, the implementation of digital technology may become more effective as faculty members make a parallel shift in their philosophical understanding of what it means to be present—perhaps even moving towards a posthumanist understanding.

### Question 2: How might University Teachers Think about Themselves and Their Role Now? How do You Think Their Identity has Changed/Must Change?

#### Luca Botturi (E1)

I see three points of change:

1. Those teachers who engaged seriously and curiously with their educational mission during the pandemic have developed now a more balanced view of their relationship with technologies. ‘Dystopian’ (so to say) teachers have discovered that technologies do not spoil or hinder education; on the contrary, they allowed teaching and learning to continue also at distance, they are allies. ‘Evangelist’ teachers have experienced that when you move to fully online many things are missing, not only in socialization, but also in actual teaching (e.g., catching the ‘teachable moments’ or collecting evidence of learning). *Teachers’ relationship with digital technologies is now based less on ideas and ideologies and more on experience – and this is always a good thing*.
2. Teachers discovered that they are not just ‘knowledge dispensers’ for their students: they are also *organizers of events in which students meet with a purpose*, and they are adults or experts that students can meet and interact with also informally. Teachers are the co-creators of the academic learning space, a space that at distance tended to dissolve. Their social role is ensuring that such places as universities continue to properly exist beyond their certification (paper mill) function. I have also the feeling that many teachers (re-)discovered that students are for them important social and intellectual stimuli.
3. Finally, I think teachers realized that disruptive change can and will happen. No perfect routine is forever. This has become a possibly uncomfortable part of their identity: *being a teacher is not keeping up some specific instructional form but adjusting to the ever-changing and diverse needs of the students.*

#### Peter Goodyear (E2)

First, I think there’s a stronger sense that the university teacher’s role involves quite a complicated form of *caring* – for students, but also for colleagues. I think many teachers have exhibited aspects of this for a long time, but the disruptions around Covid-19, the increased visibility of parts of students’ lives, and the breakdown of some settled assumptions about the limits of care have all combined to make this aspect of teachers’ work more salient, and more talked about. For example, in British HE, one once heard teachers talk dismissively about ‘spoon-feeding’ students. This term touched on a multitude of practices of care, leaving some teachers – perhaps newer teachers – uncertain about the boundaries of their roles. I think *teachers will now feel both free and obliged to talk more carefully about students’ diverse needs and situations*.

Secondly, I think we can see that the discourse around university teaching is now indicating greater seriousness about design for students’ learning, use of technologies, intended outcomes and valued practices. After the first emergency response, *there’s a greater awareness of how some extra upfront planning can save work and reduce risks later on*. Thirdly, but by no means finally, I think there’s a sharper awareness of both the *fragility and the strength of higher education*. In Australia, at least, there’s a much clearer sense of how dependent we were on revenues from international students and on the teaching work done by people hired on casual contracts. Doing something about the precarious working conditions of many teaching colleagues is now firmly on the agenda.

#### Lourdes Guàrdia (E3)

When the educational model is mainly supported by technologies, this can affect teachers’ identities and their approaches to teaching conceived as more student-centered than teacher-dominated. For example, teachers will probably use a more constructivist theory applying a variety of instructional methods in their efforts to respond to different learning styles and ways of student engagement. In this sense, technologies could support a wide range of learning activities and strategies. Then, the teachers’ role would be to encourage and promote students’ autonomy guiding and supporting their learning process.

In a technology-rich learning environment, the teacher acts as *tutor, organizer of the learning process, curator of the learning resources, motivator and project manager of students’ learning*. Furthermore, students do not necessarily need teachers when they have a ubiquitous access to online resources. Finally, the shift in teachers’ role also implies a shift in students’ role, who gradually assume more responsibility for their own learning.

#### Marguerite Koole (E4)

Many educators likely see themselves as tech-savvy, non-tech-savvy or somewhere on the continuum. Instructors who lacked a sense of self-efficacy regarding technology struggled greatly with the rapid shift online. Those already comfortable with technology adapted well and, therefore, felt successful. Lack of adequate support throughout the instructional period likely reinforced pre-existing levels of efficacy.

Informal conversations with fellow faculty members have revealed to me that some instructors who initially lacked knowledge of online instruction experienced tremendous success but had relied heavily upon institutionally based distance and instructional design experts. Galyen et al. (2021: 310) suggest that in lieu of extensive interaction with instructional design experts over several months, *teachers may benefit from cognitive apprenticeships in which they co-design and interact with design experts*, discuss design experiences with other instructors, develop an agile mindset, and embrace ‘productive failure’. These strategies, in effect, could serve to induct instructors into a community of practice—that of designers and online/blended educators rather than less helpful identities or communities based on tech-savviness.

### Question 3: How can Learning Assessment Change in the Post-Covid Era?

#### Luca Botturi (E1)

Assessment was the big unresolved issue of the emergency remote teaching period. Once again, we had the proof that assessment is the key function in learning, the lever to which everything else is connected.

We have experienced that the classic end-of-the-course test does not work at distance, despite all proctoring services, and we have been forced to look for alternatives. By doing so, we realized that new modes of evaluation can change the focus of evaluation itself, e.g., from factual knowledge to competences, or *from a one-time snapshot (the test) to the ongoing collection of evidence*. Teachers and managers were confronted with the impossibility of preserving a form which was selected because of its *efficiency* (you test many students at once), of its resistance to cheating (assessment *is* control) and of its supposed yet apparent equality.

I think that the need to rethink how we assess learning already brought positive consequences. After such experience, I expect more teachers to look for a more holistic approach to assessment, and to be able to actually *design the assessment*, and not just the questions that will appear in a taken-for-granted end-of-the-course test. Design means taking into account the actual target competences, the number and profile of the students, and considering the whole course as a potential source of evidence.

#### Peter Goodyear (E2)

There has been a great deal of talk – more heat than light sometimes – about academic integrity, cheating, proctoring and so on. In some ways I think making provision for assessment during the shift to ERT was at least as complicated as setting up online classes, and was fraught with previously implicit assumptions about what it is reasonable to ask students to do and about how students will behave. Some universities got this wrong, it seems. They prioritized defending against cheating over the well-being of their students. Of course, in some cases this is very important to get right – in areas involved with professional qualification, for example, or where certifying students as safe to practice is involved. But in other cases, I think we saw concerns about cheating being inflamed by companies selling proctoring solutions.

One strong line of argument that emerged during ERT was that universities should be more relaxed about assessment, assess less, and be more realistic about what capabilities can be assessed and how. I would like to think that the discussions and controversies about assessment will enable some more serious and flexible thinking about assessment practices generally. Coincidentally, there has been a lot of good work coming out from some of the leading researchers and innovators in assessment and feedback – on areas like feedback literacy, peer assessment, assessment involving comparison with other students’ work, internal feedback, and so on. I think we might find the post-Covid times will be *more open to more intelligent and diverse arrangements for both formative and summative assessment, and more open to including students in the discussion*.

#### Lourdes Guàrdia (E3)

For many teachers, virtual assessment in the Covid-19 pandemic context has been a nightmare because it has been difficult for them to think of another way to assess that is not based on face-to-face or traditional exams. On the other hand, the replacement of face-to-face examinations by virtual assessments has raised issues regarding students’ identity. The solution proposed by the majority of campus-based institutions is the eProctoring technology, which allows the digitalisation of the assessment using artificial intelligence and facial recognition to verify a student’s identity, but also monitoring their activity during the online examination or assessment, maintaining the same integrity and quality as when they do face-to-face exams. However, it is not easy to implement such technology due to students’ data protection issues, so the challenge is still open.

Probably there is still no consensus about how a potential assessment transformation in the post-Covid era should be: to see assessment as continuous and formative, as a more evidence-based process, as an immersive experience for learners, allowing follow-up of the students’ learning progress at any time, remotely and combining synchronous and asynchronous modalities. *Replacement of examinations with continuous assessment is certainly an option*, because the emphasis is not only to focus on what the student knows, but on what the student is *able to do* and on the *value of transferable and essential skills*, which are in fact the skills and competencies that any student should develop.

#### Marguerite Koole (E4)

In expanding conceptions of engagement and learning, a greater array of evidence for students’ interaction and understanding is helpful. Academic integrity, particularly the integrity of online examinations, became a major concern for post-secondary institutions around the world. Online examination invigilation tools were implemented with mixed results. Many institutions eventually began to discard these tools. So, what happened? These tools use algorithms for the surveillance of students by recording their activities such as the websites they visit, their body and facial movements, and the computer tools they use (Swauger 2020). Not only is there a lack of evidence that the tools effectively detect cheating (Swauger 2020), but students complained of privacy violations, delays in responses from proctors, technical and connectivity issues, increased stress, interruptions from pets, roommates, family members when taking exams remotely (Flaherty 2020), and even false positives (i.e., the system incorrectly indicates a student was cheating).

A question that has emerged from the experiment with online examination proctoring tools is, why do universities continue to rely upon traditional examinations? Schlesselman (2020) recommends *increased flexibility in assessment as well as additional formative assessment activities*. While some programs must adhere to professional standards in order to maintain certification and ensure graduates are fully qualified for high-risk jobs, other programs would benefit from focusing more on ascertaining *the quality of the learning experience itself*.

### Question 4: What Advice would You Give to Campus-based University Teachers Interested in Improving Their Students’ Self-regulation Skills? What Types of Activities can be Designed Towards This Direction?

#### Luca Botturi (E1)

During the pandemic teachers discovered that students can be (or become) more autonomous than they thought. They also experienced the importance of self-regulation and self-motivation. I think four pieces of advice can be formulated:

1. *Blend regulated activities with flexible ones*, like online ones (but there is no need to be at distance or online to propose flexible work!).
2. *Foster peer-interactions and peer-support*: activating personal networks is part of being autonomous (that does not mean do everything by oneself).
3. Allow *personalisation of curriculum*, both within a course (e.g., select one topic to study among three proposed) and within the programme (elective courses, individual endeavours, etc.).
4. Provide scaffolding to foster autonomy, rather than control over student activity; this includes offering tools for self-assessment, so that students learn to ask for scaffolding when they need it.

#### Peter Goodyear (E2)

I’ve written quite a lot about the capabilities involved in becoming an autonomous lifelong learner and the main point I would make here is that it is helpful to see the scope of these capabilities as quite wide-ranging and to understand that the development of these capabilities can be assisted through a judicious mixture of hands-on experience and direct instruction. I’m not so interested in the line of psychological research on self-regulation that casts it in narrow motivational terms – verging on claims about personality traits. Rather, I prefer to start with an analysis of actions and capabilities in (say) graduate workplaces and work backwards to the design of tasks and learning situations that are likely to help students develop and reflect on their capabilities.

For instance, successful engagement in complex knowledge work involves a capability to construct an appropriate epistemic environment: knowing how to assemble the right tools and other resources, find knowledgeable people, organise a productive sequence of knowledge-building tasks, and so on (Markauskaite and Goodyear 2017). We can think of this in terms of ‘learning to play an epistemic game’. Like learning to play tennis, it needs direct experience; it helps to play with/against people who are already good at the game, and there is a role for direct instruction and coaching.

So, as advice to other university teachers, I’d say: *focus on the kinds of epistemic games that are core to your discipline or profession and examine the capabilities that are involved in ‘playing’ them successfully*. What experiences would benefit students who need to learn to play those games? What are the rules of the game, the typical tools and instruments, the typical moves, and so on? What can you advise your students to do in advance?

#### Lourdes Guàrdia (E3)

Motivation is a key element in the success of students, and teachers play a pivotal role in this. To improve students’ motivation, teachers must know how to give students responsibility, for example through allowing them to lead learning activities, how to design work in groups facilitating social interaction that can get them excited building something together, etc. Covid-19 has shown that good learning design and guidance should help a lot to keep students on track, but also to allow them to choose topics, activities, even some assignments, providing them with a certain control that makes them more confident. *The design of authentic activities implies knowledge in the use of active learning methodologies* such as: problem solving, game-based learning, project-based and case-based learning, design thinking, inquiry-based learning, among others. These and other ‘active’ methods place the students at the centre of the learning process asking them to combine self-regulation, autonomy, creativity, collaboration, communication and other generic skills, providing a significant impact on their knowledge.

Formative assessment is another crucial aspect related to students’ motivation that should be included as a usual strategy, providing feedback continuously through activities that allows instructors to follow up with the student learning progress. As an example, ePortfolios are activities that allow alignment between feedback, reflection, and improvement, as these are based on *evidence-based learning, showcasing students’ progress throughout a course*. Another important issue is when students are working and interacting online: the use of learning analytics (LA) can support teachers to predict learners’ performance and a more personalised learning experience can be provided. LA also supports a better understanding of the effectiveness of teaching practices and guides improvements for the next course iteration.

#### Marguerite Koole (E4)

Metacognitive skills can be challenging to teach. Instead of focusing on how to directly foster self-regulation, instructors might instead consider providing an environment that provides the level of support learners need. According to transactional-distance theory, transactional distance is the ‘psychological and communications space’ between the teachers and the learners (Moore 1997: 22). *Transactional distance occurs in all educational situations including face-to-face*; nonetheless, there are strategies to reduce it. Learning environments can be designed to balance structure, autonomy, and dialogue. ‘Structure expresses the rigidity or flexibility of the programme's educational objectives, teaching strategies, and evaluation methods.’ (Moore 1997: 24).

Dialogue refers to discussions, exchange of ideas, and instructions. Autonomy refers to the extent to which the learner determines their goals, assessment strategies, pace and topics. To explain in simple terms, learners who are more autonomous require less structure and dialogue; those who are less autonomous need more structure and dialogue. To assist learners in improving their self-regulation skills, instructors can progressively modify their pedagogical approach through the semester by providing more dialogue and structure at the beginning. As the semester progresses, structure and dialogue can slowly taper off while the learners can have opportunities to take increasing responsibility for their learning.

### Question 5: Overall, What are Some Lessons that Distance/Online Learning can Teach to Campus-based University Teachers?

#### Luca Botturi (E1)

First, teaching and learning can continue even under radical changes of circumstances. A change is not only a threat, but also an opportunity for professional development.

Second, *digital technologies are not an enemy disrupting ‘humane’ education, but a potential ally for a better education*. They will be so powerful an ally that we are ready to rethink our teaching and assessment from scratch, and not just fine-tune it along the path of minimum resistance.

#### Peter Goodyear (E2)

I think the main lessons here are to do with methods, concepts and language. Many university teachers learn to teach as part of their engagement in a set of ongoing academic practices: a passing on of tradition. Unless something happens to change what they do, they teach as they were taught. Covid-19 and ERT have disturbed that. Academics are used to methodological and conceptual innovation in their *research* practices – they are not incapable of change, whatever critics outside the university may say. The disruptions associated with Covid-19 have shown that there are other educational practices available: that teachers and students now have a range of choices to make. And many teachers are realising that some of these choices are complex enough that tackling them needs the kind of design and monitoring work that they are used to doing when carrying out research.

*Design for learning benefits from some simple methods and needs some language with which to discuss and coordinate activity, across course teams, or disciplinary groups, for example*. So, I think we will see a flow of concepts and language from distance/online practice into the mainstream. In this, I hope we will see a growing realisation that putting students’ activities at the core of thinking about teaching is very helpful: *what students actually do* is what really matters, in the end.

#### Lourdes Guàrdia (E3)

The potential of an online learning model is undeniable, but only if it is properly implemented. The right approach is not to imitate what is done in a traditional model and bring it to an online environment. If the context changes, and other scenarios and opportunities arise, *the design must be consistently and accordingly transformed*.

There is much experience of quality online education that has been carried out in the last 25 years. If we take the advantage of not starting from scratch, then we should use this experience as an example: learning design is crucial for any educational purpose, but when this purpose is delivered online, design is essential. It should include: active methodologies to engage students at any time, adequate learning resources that facilitate learning at a distance and online, formative assessment that is required to provide continuous feedback to the students, guidance and support by the teacher, guaranteed accessibility and the competences required to teach and learn online.

#### Marguerite Koole (E4)

In the rush to shift to fully online teaching and learning, many instructors attempted to replicate the face-to-face modality through video conferencing technologies or lengthy pre-recorded lectures. Particularly in the case of synchronous technologies, many experienced connectivity, accessibility, and time zone challenges. A fear for distance educators is that the hastily implemented remote learning attempts were met with limited success and thereby resulted in judgments that online and/or distance learning is inferior to the face-to-face classroom. Sadly, few post-secondary instructors were familiar with the wealth of distance education literature.

Early into the pandemic, Hodges et al. (2020) referred to traditional distance education theories and practices, which certainly offers a starting point for instructors who wish to free themselves from the limitations and frustrations of replicating face-to-face instruction. The most important advice that I would offer to instructors is derived from equivalency theory: that *face-to-face and online learning need not be identical but equivalent in pedagogical value and learning outcomes* (Simonson et al. 2019).

## Discussion

As Hodges et al. (2020), Nordmann et al. (2020) and Xie and Rice (2021) point out, emergency remote teaching (ERT) during the Covid-19 pandemic has little in common with carefully planned and developed online education. Online teaching is not ordinary teaching and even less ordinary teaching through a computer. It requires careful design and consideration of various components, which need to be thought about in advance given the necessity to anticipate learners’ needs and expectations (Rapanta and Cantoni 2014). On top of that, emergency remote teachers had to face unexpected human factors that are sometimes neglected in HE, such as challenges related to learners’ motivation, socio-emotional distance, socio-economic gaps and cultural isolation. And as Schatzki (2021) points out, teachers generally have underestimated the importance of space, and the other material (and digital) dimensions of social life, in structuring educational practices. In the face of the unpreparedness of the educational world to respond to this crisis (UNESCO 2020), educators from all grades and contexts were called to take the learning situation in their hands, rethinking, reassessing and remodelling their pedagogical practices.

Even though ERT cannot be considered to share the same processes and procedures as online education, also given the lack of administrative support and technological infrastructure, it opened the path to initial digital teaching experiences. Given the rich array of possibilities within what can be broadly called ‘teaching and learning with technologies’, these initial hints of digitalisation can easily give a place to innovative and effective blended or ‘simply’ technology-enhanced forms of teaching and learning. However, as our expert interviews showed, for this to be possible, several aspects shall be taken into consideration such as flexibility, empowerment, professionalisation and strategic decision making.

### Flexibility

The design of the learning environments must be flexible, in the sense that it is consistently transformed according to the contextual learning conditions, either being conducted face-to-face or delivered remotely, or with regard to students’ needs and expected learning outcomes. Flexibly designed learning environments allow space for personalisation of the curriculum. This can be done through giving space to students to have a say in their own learning process, for example with a greater variety of courses, in different modalities and implementing different methods. At the same time, an increased personalisation implies a greater flexibility, as the instructional methods must be continually adjusted to learners’ level, interests and needs.

### Empowerment

Granting students a voice and a place in their learning process leads to a greater sense of responsibility, which in turn, if assumed by the students (and experience tells it will), can lead to a greater motivation and self-regulation. For this to take place, students’ potential must be explored not only on basis of what they want, but also and mainly on the basis of what they need and do. For this, monitoring students’ progress through continuous, evidence-based assessment methods is an important asset, and it is a wiser investment than proctoring systems to make exams ‘safe’. Instead of controlling what students do, we must create the right conditions and opportunities for them to do better.

### Professionalisation

Professionalisation refers to teachers’ and students’ attitudes within learning situations, and also to the curriculum designs that help create those situations. For teachers to behave like professionals, and therefore to be considered successful teachers, their subject matter, didactic and pedagogical competence is not enough. They must also have the critical-reflective attitude to know how and why they do what they do, and the systemic competence to adapt to changing circumstances (Zierer 2015). Adding OLT to this set of competences implies an even greater preparedness on the part of teachers, to be able to ‘hear’ and ‘see’ how the learners respond to the learning situation and whether their response is sufficient, or the activity/design must be adapted. The same applies to assessment activities, which also need to be adequately and meaningfully designed, taking into consideration both the essential, transferable skills and also the specific discipline-related competences, i.e. the ones required by young graduates in order to be able to ‘play the epistemic games’ of their professional areas.

### Strategic Decision-making

Faculty teachers are not always the ones who make the decisions regarding curriculum and assessment design. Careful strategic planning on the part of the HE institutions is now more necessary than before. This planning must include and consistently connect decision making at, at least, the three levels as detailed below^1^:

#### Macro-Level Strategies

Macro-level strategies refer to all type of institutional organisation and communication processes that influence the top-down and bottom-up flow of information and, ideally, sharing of knowledge. A clearly communicated strategy presupposes a shared language and vision of what should and should not be done in order to move forward. It also requires a certain stability of work conditions in terms of the stakeholders involved, so that decisions taken are put forward in a smoother and more integrated way. For example, if a university opts for investing in faculty’s professional development, it should also opt for less precarious employment among university teachers, so that development makes sense in a more socially-situated, holistic, identity-nurturing manner, rather than as an isolated, fragmented, goal-outcome paradox.

#### Meso-Level Strategies

Meso-level strategies refer to formal and informal synergies between stakeholders directly related to the teaching and learning process, and which take place outside the classroom. As part of these strategic synergies, the experts mention the importance of coordination activities across course teams, disciplinary groups and between teachers and instructional designers, when the latter are available (and if they are not, this is maybe something for the university to consider as a macro-level, long-term planning strategy). The emergence of cognitive apprenticeships or even communities of practice among educators and designers alike, which happened informally to a great degree during the pandemic, can now be strategically cultivated and sustained in the post-Covid era (see Kelly et al. 2016, for accounts of networked professional learning among schoolteachers).

#### Micro-Level Strategies

Micro-level strategies include pedagogical strategies and teaching methods that bridge the ‘distance’ between teachers and students, with distance referring either to the remote aspects of teaching and learning and/or to the transactional distance present in all types of learning environments. These strategies include implementation of active learning methodologies, in which students adopt a central role in the learning process in the sense of being engaged in activities that make them *walk the walk* and not just *talk the talk* (Keys 2005). In an active teaching–learning process, mutuality and shared responsibility are desirable, rather than one of the two parties, either teachers or students, being more important than the other.

Another strategy is the blending of different instructional approaches that promote a flexible and continuous assessment of the learning activity, rather than sticking to one method or two and following them as an orthodoxy. Blending of instructional approaches does not only refer to combining in situ with distance learning; it primarily includes a blending of multimodal activities, tools, and methods to achieve the desired learning outcomes (Picciano 2009). What will be blended and how highly depends on how students welcome such approaches throughout the course and across semesters.

**Fig. 1:**
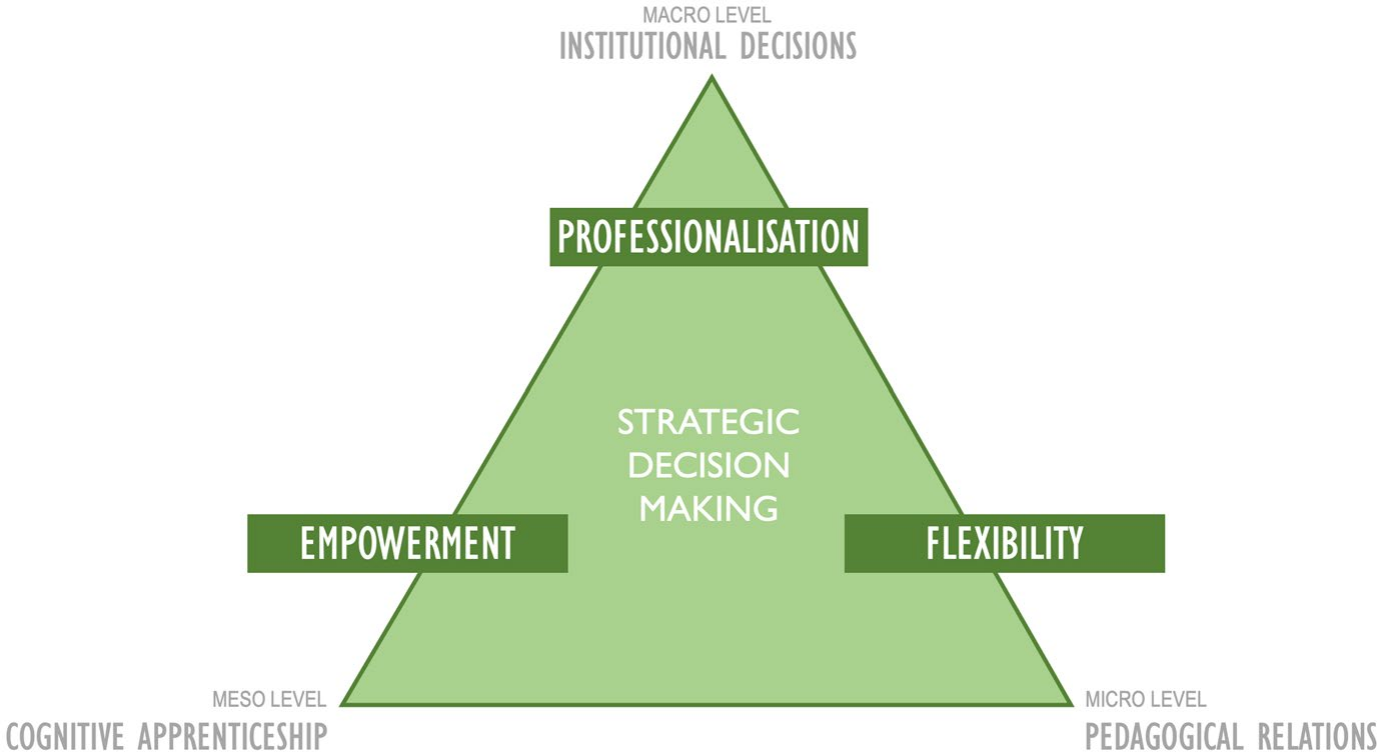
Learning design components for the post-Covid era universities

Teachers must always be willing to design and redesign their syllabi and materials to make sure that they adapt to learners’ needs, contexts and capacities. In addition, environments that promote peer-to-peer and teacher-student interaction help achieve the balance between course structure and student autonomy. Especially group work with concrete and scaffolded tasks to achieve is highly beneficial for students, as it allows them to consolidate their knowledge through interaction with peers, regulate themselves as part of a group and grow as members of a democratic society where various — and often contradictory — opinions can be valid at the same time (but some more valid than others according to the information used as evidence to support them — see, for example, Iordanou and Rapanta 2021; Kuhn et al. 2016; Rapanta 2021). Figure 1 presents a synthesis of the suggested advice by the four experts.

## Towards A Digitalisation of HE or A ‘Pedagogisation’ of Technology Use In HE?

Given the uncertainty of the times, we opt for closing this sequel to Rapanta et al. (2020) with a dilemma rather than a conclusion. This dilemma, emerging from the expert answers, transcends the three levels of strategic decision making described above. Should the focus of HE policymakers, designers and educators be on the ‘hard’ or ‘soft’ skills aspects of innovative learning? In other words, does the post-Covid challenge relate to how HE institutions become more digitalised, or to how pedagogically prepared and informed HE curricula are? This echoes previous research on the transition to e-learning, where it emerged that e-learning design was often a catalyst for a deeper instructional design reflection at an organisational level (Botturi et al. 2006).

Lohr et al. (2021) suggest that students become more cognitively engaged, and therefore learn better, when they move from passive to active, from active to constructive and from constructive to interactive activities, what is known as the ICAP (interactive > constructive > active > passive) framework of digital learning. However, as constructive and interactive digital learning activities require more time and effort by the teachers, what happens at the end is a prioritisation of passive digital learning activities, knowing that these are the ones that are less beneficial for students. This paradoxical situation is the same with non-digital learning environments: although authoritative, non-interactive, recitational teaching does not lead to substantial learning benefits (Mehan 2013), many teachers opt for expository and evaluative forms of interaction with students rather than creating opportunities and ‘space’ for students to intervene and actively express and defend their viewpoints.

Designing (inter)active learning environments is, therefore, a challenge that endures. For digital learning—whether in a blended, hybrid, or online form—a number of institutional, organisational and administrative factors condition the more proximal influences on the teacher and student level, which then shape the effectiveness of teaching and learning. It is essential to address issues at all these levels (Hofer et al. 2021; Ellis and Goodyear 2019). Institutional, teacher and learner expectations must be aligned in a way that allows time and effort to be dedicated to careful course design, if results of design are to be meaningful. A carefully planned course is one that includes constructive and interactive learning activities, leading to assessable outcomes on a continuum of desired competences in the field for which the course is designed. To achieve this alignment between expectations of the different primary stakeholders involved in what can be called an educational contract, several strategies are possible, depending on the university’s requirements, resources and organisational culture (Hofer et al. 2021).

Within a context of meaningful, strategic planning, the integration of technologies as part of pedagogical innovation is essential. Now that the pandemic has violently and abruptly necessitated such innovation, it is time to rethink the practices of HE: moving towards a more harmonious integration of physical and digital tools and methods for the sake of more active, flexible and meaningful learning. We end with the words of Pope Francis: ‘Peggio di questa crisi c’è solo il dramma di sprecarla’^2^ — the only thing worse than this crisis is the tragedy of wasting it, in the sense of failing to learn from it.

## Footnotes

1 The idea of three levels is inspired from curricular decision-making literature (see for example, Roldão amd Almeida 2018) where ‘macro’ refers to central policymaking, ‘meso’ to institutional and group decision-making, and ‘micro’ to the individual implementation.

2 See https://www.vatican.va/content/francesco/it/homilies/2020/documents/papa-francesco_20200531_omelia-pentecoste.html. Accessed 15 July 2021.
